# Supplementary material for: Force and Stepwise Movements of Gliding Motility in Human Pathogenic Bacterium Mycoplasma pneumoniae
Source: Front Microbiol. 2021 Sep 24;12:747905. doi: 10.3389/fmicb.2021.747905 (PMC8498583; doi:10.3389/fmicb.2021.747905)
Supplement: Supplementary Figure 1 — Multiple sequence alignments for P1 adhesin of M129 and FH strains. The symbols “∗” “:” “.” indicate fully conserved residue, conservation between groups of strongly similar properties, and conservation between groups of weakly similar properties, respectively. [file Data_Sheet_1.DOCX]

Supplementary Material

# Supplementary Data

Supplementary Material should be uploaded separately on submission. Please include any supplementary data, figures and/or tables. All supplementary files are deposited to FigShare for permanent storage and receive a DOI.

Supplementary material is not typeset so please ensure that all information is clearly presented, the appropriate caption is included in the file and not in the manuscript, and that the style conforms to the rest of the article. To avoid discrepancies between the published article and the supplementary material, please do not add the title, author list, affiliations or correspondence in the supplementary files.

# Supplementary Figures and Tables

For more information on Supplementary Material and for details on the different file types accepted, please see [here](http://home.frontiersin.org/about/author-guidelines#SupplementaryMaterial). Figures, tables, and images will be published under a Creative Commons CC-BY licence and permission must be obtained for use of copyrighted material from other sources (including re-published/adapted/modified/partial figures and images from the internet). It is the responsibility of the authors to acquire the licenses, to follow any citation instructions requested by third-party rights holders, and cover any supplementary charges.

## Supplementary Figures

**Supplementary Table 1.** Amino acid variations of gliding related proteins in M129 and FH strains.


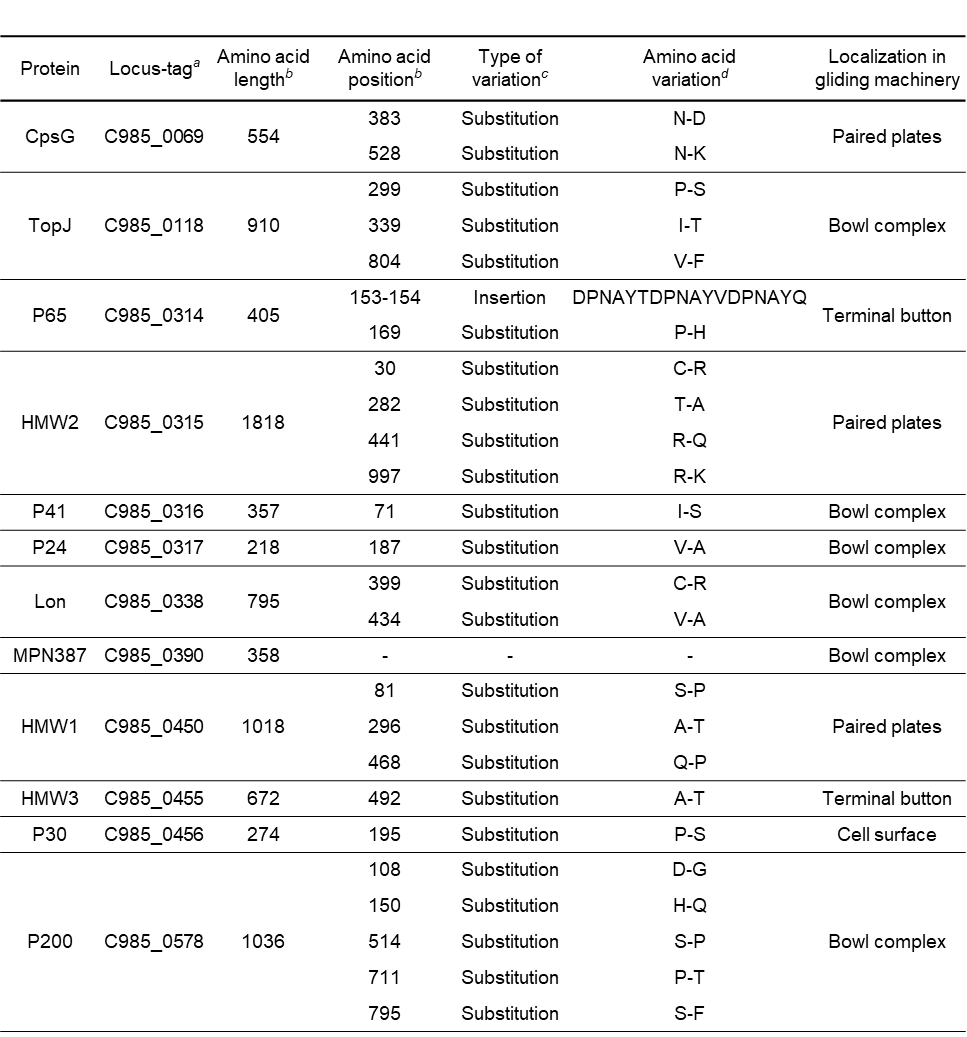


*^a^* Locus-tag is as in the sequence under accession number CP003913 for M129-B7.

*^b^* The values are for M129.

*^c^* The variations were detected in FH against M129.

*^d^* The amino acids are written the order M129-FH.

**Supplementary Figure 1. Multiple sequence alignments for P1 adhesin of M129 and FH strains.** The symbols “*” “:” “.” indicate fully conserved residue, conservation between groups of strongly similar properties, and conservation between groups of weakly similar properties, respectively.

**Supplementary Figure 2. Multiple sequence alignments for P40/P90 of M129 and FH strains.** The symbols “*” “:” “.” indicate fully conserved residue, conservation between groups of strongly similar properties, and conservation between groups of weakly similar properties, respectively.

**Supplementary Figure 3. Work performed by stepwise movements.** The scatter dot plot of works calculated from individual steps is shown with average (thick line) and standard deviation (thin lines).

**Supplementary Video 1. Stall force measurement in M129 strain.** A polystyrene bead was attached to the back end of cell body. The cell pulled the bead from trap center of optical tweezers. The video was played at 5 × speed.

**Supplementary Video 2. Stall force measurement in FH strain.** A polystyrene bead was attached to the back end of cell body. The cell pulled the bead from trap center of optical tweezers. The video was played at 5 × speed.

**Supplementary Video 3. Gliding movement of M129 strain cells.** Cells bound to the SOs-coated glass surface were observed by phase-contrast microscopy. The video was played at 5 × speed.

**Supplementary Video 4. Gliding movement of FH strain cells.** Cells bound to the SOs-coated glass surface were observed by phase-contrast microscopy. The video was played at 5 × speed.
